# A Qualitative Assessment of Studies Evaluating the Classification Accuracy of Personnel Using START in Disaster Triage: A Scoping Review

**DOI:** 10.3389/fpubh.2022.676704

**Published:** 2022-02-24

**Authors:** Uirá Duarte Wisnesky, Scott W. Kirkland, Brian H. Rowe, Sandra Campbell, Jeffrey Michael Franc

**Affiliations:** ^1^Department of Emergency Medicine, Faculty of Medicine and Dentistry, University of Alberta, Edmonton, AB, Canada; ^2^School of Public Health, University of Alberta, Edmonton, AB, Canada; ^3^J.W. Scott Health Sciences Library, University of Alberta, Edmonton, AB, Canada

**Keywords:** triage, START, mass casualty incidents, systematic review, emergency medicine, disaster medicine

## Abstract

**Background:**

Mass casualty incidents (MCIs) can occur as a consequence of a wide variety of events and often require overwhelming prehospital and emergency support and coordinated emergency response. A variety of disaster triage systems have been developed to assist health care providers in making difficult choices with regards to prioritization of victim treatment. The simple triage and rapid treatment (START) triage system is one of the most widely used triage algorithms; however, the research literature addressing real-world or simulation studies documenting the classification accuracy of personnel using START is lacking.

**Aims and Objectives:**

To explore the existing literature related to the current state of knowledge about studies assessing the classification accuracy of the START triage system.

**Design:**

Scoping review based on Arksey and O'Malley's methodological framework and narrative synthesis based on methods described by Popay and colleagues were performed.

**Results:**

The literature search identified 1,820 citations, of which 32 studies met the inclusion criteria. Thirty were peer-reviewed articles and 28 published in the last 10 years (i.e., 2010 and onward). Primary research studies originated in 13 countries and included 3,706 participants conducting triaging assessments involving 2,950 victims. Included studies consisted of five randomized controlled trials, 17 non-randomized controlled studies, eight descriptive studies, and two mixed-method studies. Simulation techniques, mode of delivery, contextual features, and participants' required skills varied among studies. Overall, there was no consistent reporting of outcomes across studies and results were heterogeneous. Data were extracted from the included studies and categorized into two themes: (1) typology of simulations and (2) START system in MCIs simulations. Each theme contains sub-themes regarding the development of simulation employing START as a system for improving individuals' preparedness. These include types of simulation training, settings, and technologies. Other sub-themes include outcome measures and reference standards.

**Conclusion:**

This review demonstrates a variety of factors impacting the development and implementation of simulation to assess characteristics of the START system. To further improve simulation-based assessment of triage systems, we recommend the use of reporting guidelines specifically designed for health care simulation research. In particular, reporting of reference standards and test characteristics need to improve in future studies.

## Introduction

Mass casualty incidents (**MCIs**) can occur as a consequence of a wide variety of events, such as those resulting from emergencies, disasters, or pandemics, and often require enhanced prehospital and emergency supports and coordinated emergency response. When MCIs cause the demand for medical care to exceed capacity, prioritization of patients shifts from treatment of the most severe casualties to an attempt to provide the best care for the highest number of victims. In these situations, medical professionals allocate priority to those who are most likely to benefit from the available resources and have the best chance of survival and recovery (1).

Created in the 1980s, the Simple Triage and Rapid Treatment (**START**) triage system was developed to be used in the event of a MCI (2), allowing responders to triage a patient in fewer than 60 seconds (s). (3). It has since become widely adopted (4, 5), especially in the United States, Canada, Australia and the Israeli-occupied territories (6). Its main goal is to appraise and identify conditions that can lead to death if not treated within 1 h by prioritizing clinical markers of respiration, perfusion, and mental status to identify impaired breathing, severe hemorrhage, and head injury. Responders employing START evaluate victims assigning them to one of four triage categories: deceased/expectant (black), immediate (red), delayed (yellow), and walking wounded/minor (green). Inaccuracies in correctly evaluating victims to a START triage category can result in either under-triage (not recognizing that victims could likely benefit from urgent medical intervention) or over-triage (in which valuable resources are used prematurely or unnecessarily). An effective triage tool should have a high sensitivity to minimize the occurrence of under-triage, but should not undermine specificity to prevent the occurrence of over-triage. Sensitivity and specificity can be determined using the rate of appropriately assigned clinical priority levels for victims of a MCI against a reference standard.

The highly stochastic nature of MCIs, as well as the complexity of subsystem interactions, makes simulation one of the best strategies for preparing individuals and health systems to develop the most efficient procedures. START is often utilized in simulation studies employing a variety of MCI scenarios assessing, for example, the impact of educational interventions, the effect of different simulation technologies, or its performance in comparison to other triage systems (7–9). A common element in these studies is the evaluation of the ability of participants to apply START in view of various outcome measures of classification accuracy. This is done to assess whether victims are being triaged to the appropriate triage category. Thus, observing simulation strategies employed in different studies and whether participants/trainees are triaging appropriately using one of the most adopted triage systems is an important step to advance studies using simulation in the field of disaster medicine.

Despite the widespread utilization of START across the literature, there was just one published synthesis of the classification accuracy of START. In this recently published systematic review it was found that the accuracy of START is insufficient to serve as a reliable disaster triage tool (10); however, it was noted that the included studies varied considerably in terms of the use of true vs. simulated MCIs, the implementation and conduct of the simulations, as well as the assessors applying the START triage system. While beyond the scope of the systematic review (10), a description of the characteristics of the simulations in which START accuracy is assessed is essential for several reasons (11–15). First, it can reveal nuances of the interaction of both (simulation techniques and triage systems) and recommend adaptations (if necessary). Second, reproducibility of findings can also be considered. Thus, the research question directing this scoping review is: What is known about simulation studies of MCIs assessing the classification accuracy of the START triage system? The purpose of this scoping review is two-fold: first, to explore the existing literature related to the current state of knowledge about simulation strategies of studies assessing the classification accuracy of the START triage system; second, to consider implications for further research.

## Methods

This scoping review was conducted following the methodological framework described by Arksey and O'Malley (16) including: identifying the design and search question; searching for relevant studies; selection of studies; charting the data; and finally, collating, summarizing and reporting the results. The methods of this study were enhanced by the recommendations of Levac, Colquhoun and O'Brien (17), which include connecting the research question to the purpose, ensuring that practicality does not limit the findings of the study, and identifying practical implications of the review. We did not engage in the optional stage 6—consultation with the community—in this current study, although such consultation may form a part of future knowledge translation. This scoping review followed the Preferred Reporting Items for Systematic Reviews and Meta-Analyses for Scoping Reviews (PRISMA-ScR) (18) (see Supplementary Material 1).

### Search Terms and Strategies

Following an initial search to identify publications on the topic, a health sciences librarian (SC) developed a search of nine electronic databases including OVID Medline, OVID EMBASE, OVID Global Health, EBSCO CINAHL, Compendex (Engineering Village), SCOPUS, Proquest Dissertations and Theses Global, Cochrane Library, and PROSPERO. The search strings for each database was adjusted appropriately for different databases and included controlled vocabulary and keywords for three concepts: (1) START, (2) triage and (3) mass casualty. The search was conducted in March 2020 and databases searches were limited from 1983 to present. No other language or publication limitations were applied. Detailed search strategies are available in Supplementary Material 2. Search results were exported to RefWorks citation management system (ProQuest, LLC, Ann Arbor, USA) and the Covidence systematic review program (Veritas Health Innovation Ltd, Melbourne, Australia).

To identify additional studies, a search of the gray literature was conducted in May 2020 which included Google Scholar, Controlled-trials.com, a forward search of the included studies using Web of Science SCOPUS, and a search of the references of included studies and relevant reviews. In addition, recent conference abstracts (2017–2020) from *Canadian Journal of Emergency Medicine, Academic Emergency Medicine*, and *Annals of Emergency Medicine* were searched. Non-English language papers were translated first *via* native speaker, or using Google Translate if a native speaker was not available.

### Study Screening and Selection

Following the removal of duplicates, the title and abstract of all articles identified in the search were reviewed by two independent reviewers (UDW and SWK) to identify potentially eligible studies based on the inclusion criteria. Once identified, the full-text of all studies classified as potentially eligible were reviewed by two reviewers (UDW and SWK) in duplicate. Decisions of inclusion or exclusion were made independently based on pre-defined inclusion criteria.

To be eligible for inclusion in the current scoping review, studies had to utilize the START triage system either in a true or simulated MCI scenario for the triage of adult victims. Studies that strictly used a modified version of START were not eligible. In addition, studies had to report outcomes related to the classification accuracy of START (i.e., accuracy, over-triage, under-triage, sensitivity, specificity) to be included. Studies were required to consist of a single cohort or multiple groups as long as at least one of the study cohorts were triaged using the START triage system. Non-experimental studies including case-reports, case-series, reviews, and editorials/opinion pieces were excluded.

Reasons for exclusion were documented. Multiple reports of the same study were collated so that each study, rather than each report, was the unit of review. Disagreements regarding study inclusion were resolved via a third-party adjudication (JMF). The results of the search, screening, and selection are reported in full in a PRISMA flow diagram (19).

### Charting, Collating, and Reporting the Results

For studies included in the review, pre-specified outcomes were extracted onto standardized forms in Microsoft excel. Data were extracted independently by at least two of three reviewers (JMF, SWK, UDW). Disagreements were settled via discussion between the reviewers and any conflicts that could not be settled were mediated via third party adjudication (BHR, JMF). The primary outcome of interest was the summary of the methods employed to develop the MCI real or simulation study in which START was applied. As such, information regarding the nature of the simulated MCI, how the simulation was implemented, who conducted the assessments, education/training of assessors, and the triage process was collected. Additional extracted outcomes included study characteristics, reporting of classification accuracy outcomes, and details regarding the reference standard. Definition of type of MCI was based on standard definitions (20).

### Study Analysis

The heterogeneity in study methods and reported findings required a narrative approach to synthesis. Findings were grouped into themes after careful reading of the final selected publications by two reviewers (SWK, UDW). These groupings were determined in relation to the research question, and in consideration of logical presentation of the findings to a diverse audience of stakeholder readers (researchers, policy developers, educators, etc.). Face validity of the themes was established by a physician specialized in emergency and disaster medicine (JMF) and a physician specialized in emergency medicine and research synthesis (BHR). This process resulted in themes that were derived from the intended scope of the study, and included the reviewers' interpretation of the data. Thematic analysis was developed using the Lancaster University Guidance on the Conduct of Narrative Synthesis in Systematic Reviews (21). Variable labels included in the studies were extracted as “themes” in the same way as conceptual themes are extracted from qualitative research (21). Development of themes was influenced by the theoretical and disciplinary lenses of emergency medicine.

## Results

**Figure 1:**
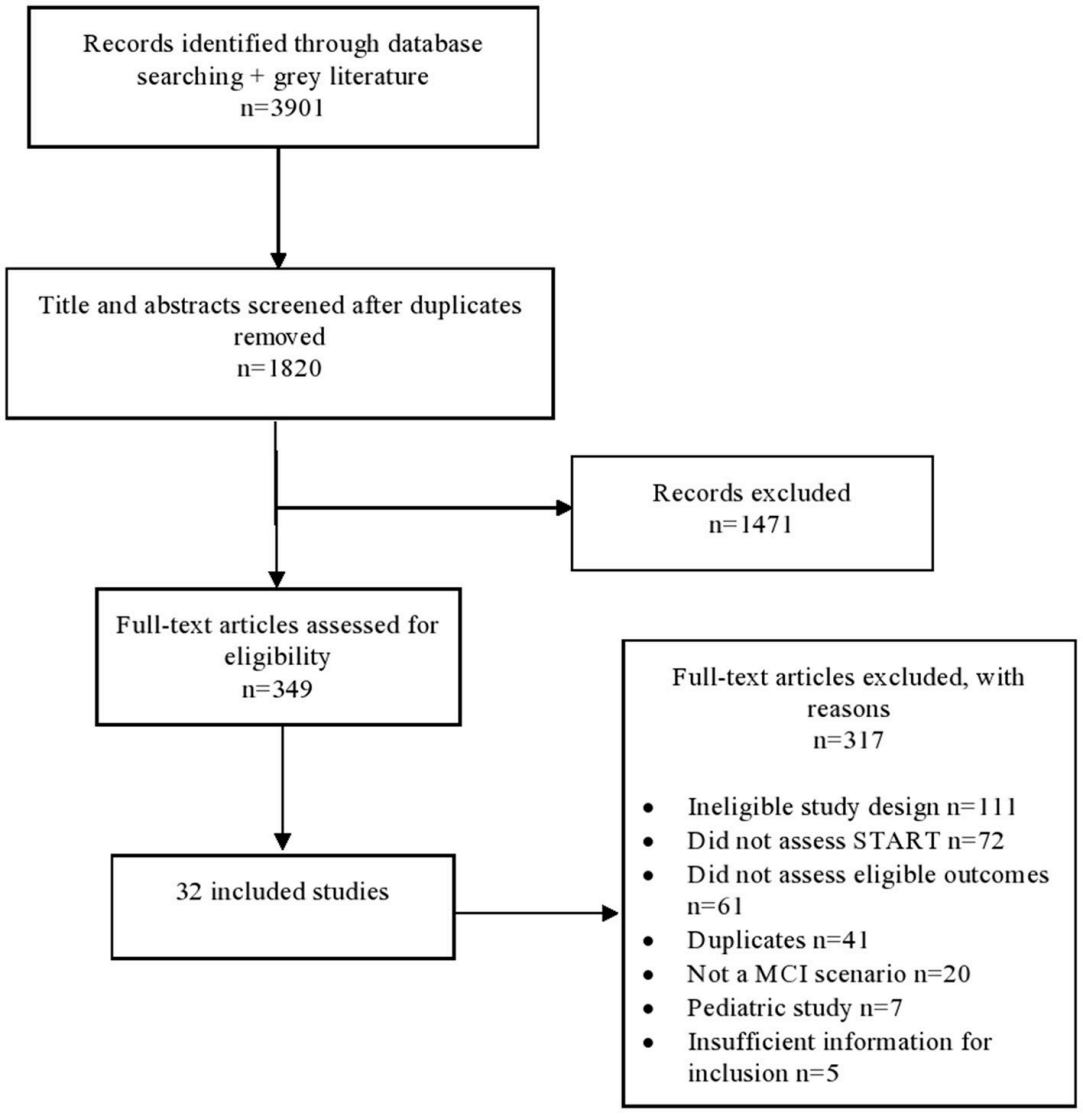
Literature search flow diagram.

After removing duplicates, the literature search yielded 1,820 citations. Following the screening of titles and abstracts, 349 publications were identified as potentially relevant. Ultimately, full-text screening resulted in the inclusion of 32 studies involving 37 cases/simulations in the review. The PRISMA flow chart of study selection is presented in Figure 1.

### Descriptive Summary of the Studies

From the 32 included studies, 30 were peer-reviewed articles, one was a conference abstract (22), and one was a master's thesis (23). The included studies were published between the years 2005 and 2019, with 28 published in the last 10 years (i.e., 2010 and onward). Studies originated from 13 countries; the United States of America (*n* = 12), Italy (*n* = 5) and Canada (*n* = 4) accounted for the majority of them. Most studies were published in English, with the exception of two (24, 25).

Research designs of included studies consisted of five randomized controlled trials (26–30), 17 comparative non-randomized studies (8, 9, 22, 25, 31–43), eight quantitative descriptive studies (7, 24, 44–49), and two mixed-method studies (23, 50). Twenty-two studies did not report their source of funding (6–9, 22, 23, 26, 31, 32, 35–42, 44, 45, 47, 48, 50) and 12 studies did not mention or acknowledged any potential conflicts of interest among the study authors (9, 22, 23, 26, 29, 32, 38, 39, 41, 43, 44, 47). Six studies did not report any study limitations (8, 24, 26, 39, 44, 49).

Together, these studies involved 3,706 participants conducting triaging assessments involving 2,950 victims. Participants conducting the triage assessment were nurses, physicians, pharmacists, emergency medical technicians, paramedics, first responders, firefighters, non-medical personnel, as well as students from different medical areas, such as paramedic, nursing, medical and various levels of training. The majority of the studies (*n* = 25) did not specify whether the participants conducting the triage assessment had prior experience with real or simulated disaster events. Tables 1, 2 presents a descriptive summary of included studies that align with the objective of the scoping review.

**Table 1:** Descriptive summary of the studies included in this review.

**References and country**	**Aim of the study**	**Study design, participants (assessors and victims, at baseline)**	**Key findings**
Arshad et al. (35), USA ✤	Determine if modification of the START system by the addition of an Orange category would reduce over- and under-triage rates in a simulated mass-casualty incident exercise.	• Quantitative non-randomized comparative study • Assessors: firefighters and paramedics (*n* = 1,457) • Assessors prior experience with MCI: not reported • Victims: computer-based cases (*n* = 30)	• The FDNY-START system may allow providers to prioritize casualties using an intermediate category (Orange) more properly aligned to meet patient needs, and as such, may reduce the rates of over-triage compared with START. • Overall correct accuracy rate was 91.2% of cases using FDNY-START whereas non-FDNY-Eagles providers correctly triaged 87.1% of cases using unmodified START
Badiali et al. (26), Italy ✤	Address whether “last-minute” START training of nonmedical personnel during a disaster or mass-casualty incident would result in more effective triage of patients.	• Quantitative randomized controlled trial • Assessors: nonprofessional first responders (*n* = 400) • Assessors prior experience with MCI: not reported • Victims: paper-based cases (*n* = 30)	• Even a “last-minute” training on the START triage protocol allows nonmedical personnel to better identify and triage the victims of a disaster or MCI. • The START group correctly triaged 94.2% of their patients, as opposed to 59.83% of the non-START group (*P* < 0.01). • Under- and over-triage were, respectively, 2.73% and 3.08% for the START group versus 13.67% and 26.5% for the non-START group. • The non-START group had 458 “preventable deaths” on 6000 cases because of incorrect triage, whereas the START group had 91.
Bolduc et al. (31), Canada ✤	Compare both accuracy and speed (triage time) of computer-based (electronic) to traditional paper-based (manual) START triage during a mass-casualty incident in a hospital setting.	• Quantitative non-randomized comparative study • Assessors: paramedics (*n* = 2) + medical doctors (*n* = 2) + registered nurses (*n* = 2) • Assessors prior experience with MCI: not reported • Victims: actors (students from an undergraduate health science program, *n* = 30)	• No significant difference in accuracy of triage when comparing electronic and manual methods, regardless of triage provider type or acuity of patient presentations
Buono et al. (22), USA ✦	Evaluate the accuracy of triage using an embedded algorithm in a wireless electronic system compared to traditional methods of triage.	• Quantitative non-randomized comparative study • Assessors: professional emergency responders (*n* = not reported) • Assessors prior experience with MCI: not reported • Victims: Unclear (*n* = 100)	• The control manual group had a 73.7% (CI: 56.9–86.6%) accuracy when compared to the gold standard. • The WIISARD-PDA group had a 72.2% (CI: 46.5–90.3) accuracy and the WIISARD-iTag group had a 67.8% (CI: 47.6- 84.1%) accuracy when compared to the gold standard (*P* = 0.09). • There was no significant difference in accuracy between the 3 methods of triage acuity determination in our MCI drill.
Challen and Walter (34), England ✤	Assess the predictive power of three different triage systems using data from an actual mass-casualty incident (the London bombings of 7th July 2005).	• Quantitative non-randomized comparative study • Assessors: Unclear (*n* = not reported) • Assessors prior experience with MCI: not reported • Victims: victims of a real mass-casualty incident (*n* = 208)	• The triage systems performed identically in identifying the critically injured, with sensitivity 50% and specificity 100% if using only the highest priority, or sensitivity 75% and specificity 99% if using the top 2 priority groups.
Crews (23), USA ^†^	Evaluate the efficacy of START triage during actual mass-casualty incidents and full-scale MCI exercises.	• Mixed-methods study • Assessors: first responders (*n* = not reported) • Assessors prior experience with MCI: not reported • Victims: victims of a real mass-casualty incident (*n* = 36) + actors (*n* = 113)	• Data analysis from actual incidents and exercises confirm that “just-in-time” training does increase the accuracy of the START triage model used from 42 to 73%.
Curran-Sills and Franc (37), Canada ✤	Compare emergency department triage nurses' time to triage and accuracy of a simulated mass-casualty incident population using a computerized version of CTAS or START systems.	• Quantitative non-randomized comparative study • Assessors: ED triage nurses (*n* = 20) • Assessors prior experience with MCI: yes (*n* = 5) • Victims: paper-based cases (*n* = 9)	• The cumulative triage accuracy for the cCTAS and START tools were 70/90 (77.8%) and 65/90 (72.2%), respectively. • The percent difference between cumulative triage was 6% (95% CI −19-8%).
Djalali et al. (48), Italy ✤	Test the association between the level of preparedness and the level of response performance during a full-scale hospital exercise	• Quantitative descriptive study • Assessors: hospital staff (*n* = not reported) • Assessors prior experience with MCI: not reported • Victims: Unclear (*n* = 61)	• The preparedness of the chosen hospital was 59%, while the response performance was evaluated as 70%. • The hospital staff conducted START triage while they received 61 casualties, which was 90% correct for the yellow group and 100% correct for the green group.
Ellebrecht et al. (25), Germany ✤	Analyze the assigned triage level of casualties and compare paramedic's performance.	• Quantitative non-randomized comparative study • Assessors: paramedics (*n* = 25) • Assessors prior experience with MCI: not reported • Victims: actors + mannequins (*n* = 559)	• Overall correct accuracy rate was 81.5%. • Percentages of inappropriately assigned triage levels ranged from 0% to 60%. A conspicuous finding was the discrepancy between fire brigade paramedics (12.3%) and other emergency services paramedics (38.5%) but the low number of cases in the study should be taken into consideration.
Ersoy and Akpinar (47), Turkey ✤	Examine the accuracy of triage decision-making among emergency physicians using a multiple casualty scenario.	• Quantitative descriptive study • Assessors: emergency physicians (*n* = 128) • Assessors prior experience with MCI: yes (*n* = 65)	• Overall accuracy rate ranged from 83.6 to 90.0% for four immediate casualties, 26.4 to 78.2% for seven urgent casualties, 70.9 to 91.8% for four delayed casualties, and 82.7 to 97.3% for two dead cases. • Emergency physicians tended to under-triage patients • Personal and professional characteristics were found to be statistically significant in five cases (*p* < 0.05).
Ferrandini-Price et al. (33), Spain ✤	Determine the e?ciency in the execution of the START triage, comparing virtual reality to clinical simulation in a mass-casualty incident.	• Quantitative non-randomized comparative study • Assessors: emergency and special care nursing master's students (*n* = 67) • Assessors prior experience with MCI: not reported • Victims: actors (3rd year students from the superior school of dramatic arts) + virtual reality cases (*n* = 20)	• No significant differences between the clinical simulation with actors group (88.3% [SD = 9.65]) and the virtual reality simulation (87.2% [SD = 7.2]) • Overall triage rate was 87.65% (SD = 8.3)
Ingrassia et al. (42), Italy ✤	Test a new disaster simulation suite evaluating its application during the same type of full-scale exercise on two different occasions.	• Quantitative non-randomized comparative study • Assessors: emergency department physicians (*n* = 36) • Assessors prior experience with MCI: yes (*n* = 18) • Victims: actors (*n* = 135)	• No differences were found as regards triage or prehospital treatment accuracy. • No usability problems arose during either simulation. • Trained physicians were faster than non-trained physicians in dispatching the victims from scene to hospital [median (interquartile range) times, 67.5 (50.0–111.0) vs. 145.0 (110.0–150.0) minutes, *P* < 0.001] • Trained physicians also treated and discharged more patients in the emergency department (32/38 vs. 14/31, *P* < 0.001) and performed better on command-and-control items (31/44 vs 17/44 for trained and non-trained players respectively, *P* < 0.05).
Ingrassia et al. (40), Italy ✤	Develop a core curriculum of disaster medicine centered on blended learning and simulation tools	• Quantitative non-randomized comparative study • Assessors: medical students (*n* = 524) • Assessors prior experience with MCI: yes (*n* = 37) • Victims: computer-based cases (*n* = 30)	• The blended approach and the use of simulation tools were appreciated by all participants and successfully increased participants' knowledge of disaster medicine and basic competencies in performing mass-casualty triage.
Ingrassia et al. (27), Italy ✤	Explore the ability of virtual reality simulation, compared with live simulation, to test mass casualty triage skills, in terms of triage accuracy, intervention correctness, and speed to complete triage, of naive medical students using the START triage algorithm in a simulated mass-casualty incident scenario and to detect the increase in this expertise after a brief learning session on mass casualty triage.	• Quantitative randomized controlled trial • Assessors: medical students (*n* = 56) • Assessors prior experience with MCI: no (*n* = 0) • Victims: actors (3rd year medical students, *n* = 10)	• No significant differences in START triage accuracy when comparing virtual reality and live simulation. • Training could improve the ability to correctly categorize patients.
Izumida et al. (39), Japan ✤	Propose a triage training system in which the expression of information changes according to the skill level of each trainee.	• Quantitative non-randomized comparative study • Assessors: university students and graduated school students (*n* = 12) • Assessors prior experience with MCI: no (*n* = 0) • Victims: virtual reality cases (*n* = 10)	• The results revealed the system was e?ective to implement triage quickly and accurately.
Jain et al. (28), Canada ✤	Compare unmanned aerial vehicle technology (UAV) to standard practice in triaging casualties at a mass-casualty incident	• Quantitative randomized controlled trial • Assessors: second-year primary care paramedic students (*n* = 20) + advance care paramedic students (*n* = 20) • Assessors prior experience with MCI: not reported • Victims: actors (*n* = 10)	• No significant differences in START triage accuracy when comparing UAV technology and standard practice. • One-hundred-percent accuracy was noted between both groups. • A non-clinical statistical difference in the time to completion with UAV groups was noted.
Kahn et al. (46), USA ✤	Analyzed whether START is accurate in assigning acuity levels to victims of a real train crash.	• Quantitative descriptive study • Assessors: paramedics (*n* = not reported) • Assessors prior experience with MCI: not reported • Victims: victims of a real mass-casualty incident (*n* = 265)	• No triage level met both the 90% sensitivity and 90% specificity requirement set forth in the hypothesis. • START ensured acceptable levels of red under-triage: 100% sensitive (95% CI 16% to 100%). • START ensured acceptable levels of green under-triage: 89.3% specific (95% CI 72% to 98%). • START incorporated a substantial amount of over-triage. • The Obuchowski statistic was 0.81, meaning that victims from a higher-acuity outcome group had an 81% chance of assignment to a higher-acuity triage category. • This analysis demonstrates poor agreement between triage levels assigned by START at a train crash and a priori outcomes criteria for each level.
Khan (29), Qatar ✤	Evaluate the mass-casualty incident triage skills of the medical staff like doctors and nurses at Hamad General Hospital Emergency Department.	• Quantitative randomized controlled trial • Assessors: physicians (*n* = 50) + nurses (*n* = 50) • Assessors prior experience with MCI: not reported • Victims: paper-based cases + computer bases cases (*n* = 40)	• The study results report 90% triage accuracy in the intervention group and 70% in control group with a difference of 20–30%. • The over and under triaging were 5% for both in the intervention side but 20%, 10% respectively in the control side. • The reliability also improved in the intervention group due to repeated training.
Lee and Franc (30), Canada ✤	Assess the ability to implement a two-step Emergency Department triage model with pre-triage using START, then subsequent triage using CTAS, during a mass-casualty incident using a computer-based disaster simulation.	• Quantitative randomized controlled trial • Assessors: emergency medicine resident physicians (*n* = 21) + triage nurses (*n* = 2) • Assessors prior experience with MCI: yes (*n* = 23) • Victims: computer-based cases of patients presenting to the ED due to a MCI (*n* = 174)	• No significant difference in accuracy of triage and patient flow when comparing a two-step emergency department triage model (CTAS + START) to START alone.
Lima et al. (45), Brazil ✤	Describe the teaching strategy based on the Multiple Victims Incident simulation, discussing and evaluating the performance of the students involved in the initial care of trauma victims.	• Quantitative descriptive study • Assessors: medical and nursing students and prehospital care team (*n* = not reported) • Assessors prior experience with MCI: not reported • Victims: actors (medical and nursing students, *n* = 56)	• Overall accuracy rate was 94.1% • Following the primary evaluation with the ABCDE mnemonic, all steps were performed correctly in 70%.
Loth et al. (36), USA ✤	Examine an adapted training protocol using START triage principles, which incorporated visually complex triage situations	• Quantitative non-randomized comparative study • Assessors: college students (*n* = 18) • Assessors prior experience with MCI: no (*n* = 0) • Victims: computer-based cases (*n* = 8)	• A short, directed triage training tool in improving the recognition of triage features was shown to be effective. • Those who underwent training only on patient transport and not on the adapted START triage protocol demonstrated no statistically significant between-session gaze measurement. • Subjects who underwent START triage training significantly improved in their first fixation entry time, indicating a faster recognition of salient triage features.
McCoy et al. (7), USA ✤	Evaluate the feasibility and effectiveness of using tele-simulation to deliver an emergency medical services course on mass-casualty incident training to healthcare providers overseas.	• Quantitative descriptive study • Assessors: healthcare providers including physicians, nurses and EMT/paramedics, pharmacists and educators/technicians (*n* = 32) • Assessors prior experience with MCI: not reported • Victims: virtual reality cases (*n* = not reported)	• There was significant difference in accuracy of triage when comparing providers
McElroy et al. (49), USA ✤	Describe the planning and implementation process, share results, and facilitate other regions as they conduct similar preparatory drills.	• Quantitative descriptive study • Assessors: EMS (*n* = not reported) • Assessors prior experience with MCI: not reported • Victims: paper-based cases + simulation cases (*n* = 445)	• Of the 445 transported patients, 270 (60%) were entered correctly into the state patient tracking system; 68 (25.2%) upgrades and 34 (12.6%) downgrades from scene triage categories were noted.
Mills et al. (50), Australia ✤	Compare the simulation efficacy of a bespoke virtual-reality (VR) mass-casualty incident simulation with an equivalent live simulation scenario designed for undergraduate paramedicine students.	• Mixed-methods study • Assessors: undergraduate paramedicine students (*n* = 29) • Assessors prior experience with MCI: yes (*n* = 29) • Victims: actors + virtual reality (*n* = 10)	• No significant differences were observed in accuracy in each platform. The VR simulation provided near identical simulation efficacy for paramedicine students compared to the live simulation.
Navin et al. (38), USA ✤	Evaluate the operational viability of Sacco Triage Method and to compare its performance to START.	• Quantitative non-randomized comparative study • Assessors: EMT-1 + EMT-Ps (*n* = not reported) • Assessors prior experience with MCI: not reported • Victims: actors (*n* = 20) + mannequins (*n* = 79)	• Sacco Triage Method scoring was more accurate at 91.7% than START assessments at 71.0%. • Surveyed providers preferred START to Sacco Triage Method falsely believing it to be more accurate, faster, and better able to identify the most serious patients.
Risavi et al. (8), USA ✤	Assess the effectiveness of written and moulage scenarios using video instruction for mass-casualty triage by evaluating skill retention at six months post intervention.	• Quantitative non-randomized comparative study • Assessors: emergency medical technician + emergency medical technician paramedics (*n* = 45) • Assessors prior experience with MCI: not reported • Victims: actors (*n* = 12) + paper-based cases (*n* = 12)	• No significant differences between written and moulage testing results at either initial testing or at six months. • Prior skill level did not in?uence test performance on the type of testing conducted or long-term retention of triage skills. • There was a significant decrease in performance between initial and six-month testing, indicating skill decay and loss of retention of triage skills after an extended nonuse period.
Riza'I et al. (41), Indonesia ✤	Evaluate the accuracy of triage decisions made by first-year medical students after receiving two intervention methods.	• Quantitative non-randomized comparative study • Assessors: first-year medical students (*n* = 54) • Assessors prior experience with MCI: not reported • Victims: paper-based cases (*n* = 10)	• The mean of method 2 (8.03 ± 0.72) was significantly improved for correct triage compared with the mean of method 1 (6.33± 1.63) for 54 students (*P* < 0.001). • The under-triage rate was significantly reduced (*P* < 0.001) from method 1 (2.24 ± 1.54) to method 2 (0.94 ± 0. 73). • The over-triage rate was also reduced from method 1 (1.42 ± 0.92) to method 2 (1.01 ± 0.56) (*P* < 0.001).
Sapp et al. (32), USA ✤	Evaluate the accuracy of triage decisions made by newly enrolled first-year medical students after receiving a brief educational intervention.	• Quantitative non-randomized comparative study • Assessors: first-year medical students (*n* = 315) • Assessors prior experience with MCI: no (*n* = 0) • Victims: paper-based cases (*n* = 15)	• Overall accuracy rate was 64.3%. First-year medical students who received brief START training achieved triage accuracy scores similar to those of emergency medical providers in previous studies. • The overall rate of over-triage was 17.8%, compared to an under-triage rate of 12.6% suggesting that a need exists for improving the accuracy of triage decisions in this group. • There were no significant differences in triage accuracy between subjects with and without printed materials (63.9% vs. 64.6%, *P* = 0.729) or those completing the age-variant test types (64.4% vs. 64.1%, *P* = 0.889).
Schenker et al. (44), USA ✤	Evaluate the accuracy and speed for the triage of multiple patients during a disaster drill by Emergency Medical Service personnel.	• Quantitative descriptive study • Assessors: EMS personnel (*n* = 40) • Assessors prior experience with MCI: not reported • Victims: actors (police cadets, *n* = 99) + mannequins (*n* = 31)	• Overall triage accuracy rate was 78%, exceeding data suggesting that the triage accuracy rates using different triage strategy algorithms are approximately 45% to 55%. • Contrary to expectations, the triage to transport times for the green-, yellow-, and red-tag patients were similar.
Silvestri et al. (9), USA ✤	Compare the START and SALT classifications of patients to a published reference standard category, and evaluated the accuracy of the START method applied by emergency medical services personnel in a field simulation.	• Quantitative non-randomized comparative study • Assessors: EMS personnel (*n* = not reported) • Assessors prior experience with MCI: not reported • Victims: actors + mannequin (*n* = 82)	• SALT triage system was overall more accurate triage method than START at classifying patients, specifically in the delayed and immediate categories. • In the field exercise, paramedic use of the START methodology yielded a higher rate of under-triage compared to the SALT classification.
Simoes et al. (24), Brazil ✤	Analyze the quality of pre-hospital care provided by agencies in Vitória-Espirito Santo, Brazil.	• Quantitative descriptive study • Assessors: the military fire brigade (*n* = not reported) • Assessors prior experience with MCI: not reported • Victims: paper-based cases (*n* = 40)	• Overall correct accuracy rate was 92.5% using START. • Overall correct accuracy rate was 92.5% of the cases using the mnemonic method (ABCDE), in terms of Airway; 97.5%, in Breathing; 92.5%, in Circulation; 90%, in Neurological Assessment; and 50%, in the Exhibition and Control of the Environment. • The ABCDE joint analysis showed that the service was correct in 42.5% of the cases.
Wu et al. (43), Taiwan ✤	Evaluate the effectiveness of a brief training course on (START.	• Quantitative non-randomized comparative study • Assessors: physicians (*n* = 18) + nurses (*n* = 145) + EMTs (*n* = 23) + hospital administrators (*n* = 41) + volunteers (*n* = 64) • Assessors prior experience with MCI: yes (*n* = 131) • Victims: paper-based cases (*n* = 12)	• The trainees' scores increased significantly after the training (*P* < 0.001). • Improvement (post-test score minus pre-test score) was not significantly different among the occupational groups. • Medical (physicians, nurses, and EMTs) and non-medical groups displayed similar improvement, but post-training scores were significantly lower in the non-medical participants (*P* < 0.001). • Trainees with prior triage training had higher pre-training scores (*P* < 0.05), but no significant improvement was evident in the non-medical personnel with prior triage training. • The level of performance of triage by non-medical personnel was less than optimal (post training score = 9.32), but the ability to divide casualties into minor (green) and major (yellow and red) groups was reliable.

*^D^Abstract*.

† *Master's thesis*.

*^F^Article*.

**Table 2:** Transparency of the studies.

**References**	**Funding source**	**Conflicts of interest**	**Limitations**	**Limitations reported by authors**
Arshad et al. (35)	✗	Stated	✓	• Lack of pertinent information (age, gender, years of service, training, and experience) about the comparison group.
				• Challenges of implementing system-wide changes to EMS protocols and training personnel. • Difficulty of prospective analyses in EMS systems.
Badiali et al. (26)	✗	Not stated	✗	Not reported.
Bolduc et al. (31)	✗	Stated	✓	• Single-center study.
				• Ordering of different triage modalities may have impacted triage time.
				• Simulation conducted differently between groups.
Buono et al. (22)	✗	Not stated	✓	• Small sample size.
				• Unintentionally ambiguous scenarios made triage level determination difficult.
Challen and Walter (34)	✓	Stated	✓	• There was a paucity of available documentation.
				• Data collection challenges since staff at the incident scenes were using their own tags as well as official supplies.
				• There was missing data within the medical records.
Crews (23)	✗	Not stated	✓	• Lack of previous studies.
				• Confinement of geographical region studied.
Curran-Sills and Franc (37)	✗	Stated	✓	• One group (nurses) were non-randomized.
				• Simulation was done with paper-based assessment tool, which is an oversimplification of actual triage.
				• It only includes adult victims.
Djalali et al. (48)	✗	Stated	✓	• Sample size from only one hospital.
				• Response performance indicators were limited to command and control actions.
Ellebrecht et al. (25)	✓	Stated ^*^	✓	• Limited generalizability
Ersoy and Akpinar (47)	✗	Not stated	✓	• The scale of the decisions may not reflect the real conditions that physicians encounter in their daily practice.
Ferrandini-Price et al. (33)	✓	Stated	✓	• Both groups were not comprised by the same individuals, so that there could be a variability due to the possible individual variations
				• The use of *ad hoc* test preclude authors to provide data on the efficiency of the tool.
Ingrassia et al. (42)	✗	Stated	✓	• For practical reasons treatment accuracy was evaluated only in the pre-hospital phase.
				• Although similar, the two scenarios were not identical since there were slight differences with regard to the resources available to each group.
				• The evaluation of performance indicators could be observer biased.
				• Since it was necessary to set a time limit, it is clear that the overall evaluation of the hospital response to the simulations is potentially biased by shorter simulation time.
Ingrassia et al. (40)	✗	Stated	✓	• Apart from the theoretical knowledge acquired and the increase of mass-casualty triage skills, the students were not evaluated for an improvement in other medical disaster management competencies.
Ingrassia et al. (27)	✓	Stated ^*^	✓	• Small sample size.
				• Selection bias.
Izumida et al. (39)	✗	Not stated	✗	Not reported.
Jain et al. (28)	✓	Stated	✓	• Technological challenges.
				• Small sample size.
Kahn et al. (46)	✓	Stated	✓	• The study methodology could not discern whether errors in assignment of triage categories resulted from failure of the triage algorithm as a tool or failure of emergency personnel to apply it correctly.
				• Possibly over-triage bias as researchers did observe that some of the assigned triage levels differed from what strict application of the START algorithm would have mandated.
				• The black, or “deceased,” category was not examined.
Khan (29)	✓	Not stated	✓	• Small sample size.
				• Single-center study.
				• Using only one tool or system of triage (START).
Lee and Franc (30)	✓	Stated ^*^	✓	• Logistical and technological challenges.
				• Issues during data collection.
				• Potential Hawthorne effect.
				• Unknown experience of participants with START prior to study.
Lima et al. (45)	✗	Stated	✓	• Lack of preparation of victims to act accordingly to injuries.
				• Displacement of the victims from the triage area to the canvases for care during simulation.
				• Place of collection and the limitation of the material used in the simulation to care for the victims were not well-defined for the participants as well.
Loth et al. (36)	✗	Stated	✓	• Small sample size.
				• Pictures only showed one victim at a time, which isn't realistic for an MCI.
				• This study failed to show significance for its secondary objective of improvement in triage accuracy.
McCoy et al. (7)	✗	Stated	✓	• Voluntary enrolment in the course, thus sample may not be representative of all professions.
				• Not designed as an observational-analytical study so not powered to detect differences between groups.
				• Heterogeneous group of “other” participants.
McElroy et al. (49)	✓	Stated	✗	Not reported.
Mills et al. (50)	✗	Stated ^*^	✓	• Small sample size of participants
				• Small number of patients (victims)
Navin et al. (38)	✗	Not stated	✓	• Assessment and scoring of victims were done from reading patient profile cards and not by making actual physiologic assessment.
				• Exercises assumed unlimited transport and treatment resources. • The use of mannequins slightly impacted the study.
				• The impact of the familiarity of the scene is unknown.
				• STM triage and resource management software was not tested.
Risavi et al. (8)	✗	Stated	✗	Not reported.
Riza'I et al. (41)	✗	Not stated	✓	• Small sample size.
Sapp et al. (32)	✗	Not stated	✓	• Lack of information of participants previous MCI training.
				• Limited generalizability to the general population as the study was done with medical students
Schenker et al. (44)	✗	Not stated	✗	• Not reported
Silvestri et al. (9)	✗	Not stated	✓	• Some of the volunteer victims might not have appropriately displayed their injuries on the cards they were wearing, which could account for some of the under-triage
Simoes et al. (24)	✓	Stated	✗	Not reported
Wu et al. (43)	✗	Not stated	✓	• Seniority of the participants were not taken into consideration.
				• The same written test was given before and after the training session, which may rise the concern of improvement comes from short-term practice but not learning.

*✓Reported*.

*✗Not reported*.

* *Potential conflict of interest*.

### Narrative Summary of the Studies

Thematic analysis of the charted findings led to the identification of two themes: (1) typology of simulations and (2) START system in MCIs simulations. Each theme contains sub-themes regarding the development of simulation employing START as a system for improving individuals' preparedness.

#### Theme 1: Typology of Simulations

This theme explores the common types and characteristics of simulations employed in the studies. Sub-themes include simulation technologies, simulation settings, disaster types, assessors and their training/experiences in MCI (see Table 3).

**Table 3:** Typology of simulations.

**References**	**Type of disaster**	**Simulation technology**	**Setting of MCI enactment and/or physical location assessors**	**MCI/disaster data source**
Arshad et al. (35)	• Land disaster (motor vehicle accidents)	• Computer-based (victims description)	• Unclear	Unclear
Badiali et al. (26)	• Unclear	• Paper-based (victims description)	• Unclear	Derived from a web-based platform, which clear defines how the cases were created
Bolduc et al. (31)	• Land disaster (train derailment) • Toxic release (chemical spill)	• Live simulation (actors)	• Emergency Department	Unclear
Buono et al. (22)	• Unclear	• Unclear	• Unclear	Unclear
Challen and Walter (34)	• Bomb threats/terrorist attack (shooting)	• Retrospective analysis of real mass casualty incident	• Not applicable: retrospective analysis	Medical records
Crews (23)	• Bomb threats/terrorist attack (shooting) • Land disaster (motor vehicle accidents) • Explosions (chemical explosion) • Air disaster (airplane accident)	• Retrospective analysis of real mass casualty incident • Live simulation (actors)	• Not applicable: retrospective analysis	Real MCI
Curran-Sills and Franc (37)	• Unclear	• Paper-based (victims description)	• Emergency Department	Derived from a web-based platform (www.disastermed.ca) but unclear how MCIs scenarios were created and validated
Djalali et al. (48)	• Explosions (chemical explosion)	• Unclear	• Hospital	Unclear
Ellebrecht et al. (25)	• Air disaster (airplane collision)	• Live simulation (actors)	• Airport	Unclear
Ersoy et al. (47)	• Land disaster (motor vehicle accidents)	• Paper-based (questionnaire with a MCI scenario)	• Unclear	Borrowed from another study, which was created by the study researchers
Ferrandini-Price et al. (33)	• Unclear	• Virtual reality (head mounted display) • Live simulation (actors)	• Unclear	Created by healthcare professionals
Ingrassia et al. (42)	• Structural collapse (ceiling collapse)	• Live simulation (actors)	• Unclear	Created by researchers
Ingrassia et al. (40)	• Land disaster (motor vehicle accidents)	Computer-based (electronic simulation designed using Adobe Flash)	• University campus	Unclear
Ingrassia et al. (27)	• Land disaster (motor vehicle accidents)	• Virtual reality (joystick) • Live simulation (actors)	• University campus	Derived from a web-based platform (VictimBase) but unclear how MCIs scenarios were created and validated
Izumida et al. (39)	• Unclear	• Virtual reality (head mounted display)	• Unclear	Unclear
Jain et al. (28)	• Land disaster (motor vehicle accidents)	• Live simulation (actors) • Computer-based (unmanned aerial vehicle)	• Airport runway	Real MCI
Kahn et al. (46)	• Land disaster (motor vehicle accidents)	• Retrospective analysis of real mass casualty incident	• Not applicable: retrospective analysis	Medical records
Khan (29)	• Unclear	• Paper-based (details not reported) • Computer-based (details not reported)	• Emergency Department	Unclear
Lee and Franc (30)	• Unclear	• Computer-based (SurgeSim)	• Emergency Department	Derived from a web-based platform (SurgeSim version 2.2.0) but unclear how MCIs scenarios were created and validated
Lima et al. (45)	• Land disaster (motor vehicle accidents)	• Live simulation (actors)	• University campus	Created by researchers
Loth et al. (36)	• Unclear	• Computer-based (latent images)	• University campus	Unclear
McCoy et al. (7)	• Bomb threats/terrorist attack (shooting)	• Virtual reality (broadcasting)	• High-rise office building	Unclear
McElroy et al. (48)	• Bomb threats/terrorist attack (terrorist attack)	• Computer-based (details not reported) • Live simulation (actors)	• University campus, soccer stadium and airport	Created by a private firm, but unclear how scenarios were created and validated
Mills et al. (50)	• Land disaster (motor vehicle accidents)	• Virtual reality (actors) • Live simulation (head mounted display)	• Virtual reality: Police academy's ground • Live simulation: University campus	Created by researchers
Navin et al. (38)	• Structural collapse (building collapse)	• Live simulation (actors and mannequins)	• Fire Department academy	Unclear
Risavi et al. (8)	• Unclear	• Paper-based • Moulage	• Unclear	Unclear
Riza'I et al. (41)	• Unclear	• Paper-based (details not reported)	• Unclear	Unclear
Sapp et al. (32)	• Toxic release (sarin gas)	• Paper-based (questionnaire with a clinical scenario)	• University campus	Created by healthcare professionals
Schenker et al. (44)	• Explosions (chemical explosion)	• Live simulation • Mannequins	• Unclear	Created by healthcare professionals
Silvestri et al. (9)	• Explosions (chemical explosion) • Bomb threats/terrorist attack (shooting)	• Live simulation (actors and mannequins)	• University campus	Created by researchers
Simoes et al. (24)	• Land disaster (motor vehicle accidents)	• Retrospective analysis of a simulation exercise	• Unclear	Medical records
Wu et al. (43)	• Unclear	• Paper-based (details not reported)	• Unclear	Unclear

##### Simulation Technologies

The technology employed in the delivery of simulations varied considerably across the literature (see Table 3). In a few studies, victims from MCI were re-assessed retrospectively using real mass casualty incident data (23, 34, 46) or data from a previous simulation exercise (24). In some studies, paper-based simulations were employed in which a scenario was described involving victims of a MCI and participants were asked to review and apply START (8, 26, 29, 32, 37, 41, 43, 47). Other studies employed computer-based simulations, which generally involved a multimedia-facilitated activity (28–30, 35, 36, 40, 49). Computer-based simulations varied from use of latent images to more complex software in which a series of victims of a disaster or MCI arrive to an ED or other hospital setting requiring participates to triage presenting victims *via* START. The majority of the studies required participants to partake in a live simulation exercise, of which participants are at the scene of a simulated MCI and are required to apply START to actors or manikins representing the victims (8, 9, 23, 25, 27, 28, 31, 33, 38, 42, 44, 45, 49, 50).

Within the last 6 years, studies started utilizing virtual reality, where participants usually wear a head-mounted display allowing them to have a 360° visual of images and videos (27, 33, 39, 50). Virtual reality was also used by live broadcasting a MCI scenario to participants; however, instead of wearing a head-mounted display, participants guided a person via video call (7). The guide at the scene would verbalize information needed for participants, so that they could evaluate each victim and assign them the appropriate triage category (7).

It should be noted that some of these studies applied a mixed technology approach when implementing their simulations (8, 23, 27–29, 33, 49, 50). For example, one study employed the use of unmanned aerial vehicles to allow paramedical students to survey a simulated multi-vehicular accident with live actors with moulage playing the victims (28). Other studies compared different technologies for implementing simulations such as virtual reality-based simulation vs. live simulation with actors (27, 33, 50). Two studies did not report the technology employed to perform simulation exercises (22, 48), while another study reported using moulage without specifying whether manikins or live actors were used (8).

##### Simulation Settings

Simulation exercises conducted *via* paper, computer, and virtual-reality tended to occur in hospital or university settings (27, 29, 30, 32, 36, 37, 40, 49, 50). Live simulation exercises occurred in a variety of settings including university campuses (9, 27, 45, 49, 50), airports (25, 28, 49), emergency department (31), soccer stadium (49), fire department (38), and police academy (50). Twelve studies did not specify the location of the simulation exercises (8, 22, 26, 33, 35, 39, 41–44, 47, 48).

##### Disaster Types

MCI simulations across the included studies were most frequently based on transportation disasters on land (i.e., motor vehicle crashes, *n* = 10) (23, 24, 27, 28, 35, 40, 45–47, 50), followed by bomb threats/terrorist attacks (*n* = 5) (7, 9, 23, 34, 49). The remaining studies used a variety of MCI events including chemical explosion (9, 23, 44, 48), bomb threats/terrorist attack with chemical explosion (9), toxic release (31, 32), transportation disaster on air (23, 25), transportation disaster on land with chemical spill (31), and structural collapse (38, 42). Eleven studies did not report on the types of MCI they were simulating (8, 22, 26, 29, 30, 33, 36, 37, 39, 41, 43).

The sources of the simulation scenarios varied with some studies using real events with actual clinical characteristics of the victims (23, 24, 28, 34, 46). Study researchers (9, 42, 45, 47, 50) and healthcare professionals (32, 33, 44) created the MCI events and victims, while in other studies the MCI event was retrieved from third-party databases (26, 27, 30, 37, 49), which include various MCI scenarios from which researchers can choose. The source of the MCI event, as well as the characteristics of the victims, was not reported in 14 of the included studies, and so it was not clear how the MCI scenarios were created and validated (7, 8, 22, 25, 29, 31, 35, 36, 38–41, 43, 48).

##### Assessors

Studies employed a variety of medical professionals to assess the classification accuracy of START across the literature (see Table 1). First responders/paramedics were most commonly recruited to participate in studies requiring to apply START (8, 9, 22, 23, 25, 31, 38, 44, 46, 49), with two studies specifically recruiting firefighters (24, 35). Students of various professions, including a variety of college-level (36, 39), medical (27, 32, 40, 41), nursing (45), and paramedic students (28, 50) were the second most common participants recruited to apply START. Other professionals including nurses and physicians were also recruited; however, studies tended to assess the ability of a mix of health professionals to accurately apply START (7, 29–31, 37, 42, 43, 47). Few studies compared the differences in the accuracy of START among different healthcare professionals (7, 25).

##### Experience and Training in Disaster Medicine and START

Seven studies specifically reported participants had previous experience with the START system (9, 30, 35, 37, 38, 44, 46) and 11 studies specified whether or not participants had any prior experience with MCI (27, 30, 32, 36, 37, 39, 40, 42, 43, 47, 50). Seven of the 21 studies that did not report participants' prior MCI experience also did not involve any MCI education intervention or reported whether participants were trained in MCI triage for the specific study (22–24, 34, 35, 48, 49).

Of the 22 studies that offered training in MCI prior to the simulation, 14 studies included training on START (7, 8, 25–28, 32, 36, 38, 39, 41, 43, 45, 47). Training included lecture (27, 28, 32), courses (7, 41), provision of reading materials (39) symposium (45), video presentation (8). Six studies did not specify how training was provided (25, 26, 36, 38, 43, 47). Among the 16 studies that reported to offering lectures/courses, the majority of studies reported to implementing a single course/session lasting between 5 and 1,200 mins (median: 60 min; IQR = 110 min).

#### Theme 2: START System in MCIs Simulations

This theme explores how the classification accuracy of START triage system was assessed across the different studies (see Table 4).

**Table 4:** Assessment of accuracy outcomes.

**References**	**Reported outcomes**	**Reported points**	**Reference standard**
Arshad et al. (35)	• Accuracy (total and all sub-groups)	• START	Not reported
	• Over-triage (total and all sub-groups)	• Modified START	
	• Under-triage (total and all sub-groups)		
Badiali et al. (26)	• Accuracy (total and all sub-groups)	• Non-START training	Not reported
	• Over-triage (total and black sub-group)	• START last minute training	
	• Under-triage (total and black sub-group)		
Bolduc et al. (31)	• Accuracy (total and all sub-groups)	• START manual	Expert opinion
		• START electronic	
Buono et al. (22)	• Accuracy (total)	• START (WIISARD^*^-PDA^**^)	Expert opinion
		• START (WIISARD^*^-iTag^***^)	
		• START (Control^****^)	
Challen and Walter (34)	• Sensitivity (subgroup red, subgroup red + yellow)	• START	Outcomes regard sensitivity and specificity.
	• Specificity (subgroup red, subgroup red + yellow)	• Manchester Sieve	Baxt and Upeniek criticality
		• CareFlight triage	
Crews (23)	• Accuracy (total)	• START and the total population, year 2016	Expert opinion
	• Over-triage (total)	• START and the total population, year 2017	
	• Under-triage (total)	• START and the total population, year 2018	
Curran-Sills and Franc (37)	• Accuracy (total)	• START	Expert opinion
	• Over-triage (total)	• CTAS	
	• Under-triage (total)		
Djalali et al. (48)	• Accuracy (subgroup green, and subgroup yellow)	• START	Not reported
Ellebrecht et al. (25)	• Accuracy (total, and all subgroups with exception of black)	• START	Not reported
	• Over-triage (total, subgroup yellow, and subgroup green)		
	• Under-triage (total, subgroup red, and subgroup yellow)		
Ersoy et al. (47)	• Accuracy (total and all sub-groups)	• START	Not reported
	• Over-triage (total and all sub-groups)		
	• Under-triage (total and all sub-groups)		
Ferrandini-Price et al. (33)	• Accuracy (total)	• START with clinical simulation with actors	Expert opinion
		• START with virtual reality	
		• START with both clinical simulation with actors group and virtual reality	
Ingrassia et al. (42)	• Accuracy (total and all sub-groups)	• START with virtual reality on day 1	Expert opinion
	• Over triage (green sub-group, yellow sub-group, and black sub-group)	• START with virtual reality on day 3	
	• Under triage (green sub-group, yellow sub-group, and red sub-group)	• START with live simulation on day 1	
		• START with live simulation on day 3	
Ingrassia et al. (40)	• Accuracy (total)	• START before learning module (pre-test)	Not reported
		• START after learning module (post-test)	
Ingrassia et al. (27)	• Accuracy (total and all sub-groups)^*^	• START with disaster medicine training in the in pre-hospital setting	Not reported
	• Over-triage (total and all sub-groups with the exception of red ED trained subgroup, red pre-hospital non-trained subgroup)^*^	• START without previous training in medical disaster management in pre-hospital settings	
	• Under-triage (total and all sub-groups with the exception of green trained and non-trained subgroup, and trained yellow subgroup)^*^	• START with disaster medicine training in the emergency department	
	^*^Accuracy, over- and under-triage of black group and subgroups were not reported, with exception of accuracy of prehospital trained.	• START without previous training in medical disaster management in the emergency department	
Izumida et al. (39)	• Accuracy (total)	• START with a novel training system	Not reported
		• START with a training system in which difficulty does not change dynamically	
Jain et al. (28)	• Accuracy (total)	• START with an unmanned aerial vehicle drone	Not reported
		• START with live simulation	
Kahn et al. (46)	• Sensitivity (green, yellow, and red subgroups)	• START	Other triage guideline
	• Specificity (green, yellow, and red subgroups)		
	• Positive predictive value (green, yellow, and red subgroups)		
	• Negative predictive value (green, yellow, and red subgroups)		
	• Positive likelihood (green, yellow, and red subgroups)		
	• Negative likelihood (green, yellow, and red subgroups)		
	• Accuracy (total)		
	• Over-triage (total)		
	• Under-triage (total)		
Khan (29)	• Accuracy (total)	• START intervention group	Not reported
	• Over-triage (total)	• START control group	
	• Under-triage (total)		
Lee and Franc (30)	• Accuracy (total and all sub-groups, with exception of black)	• START (one-step triage)	Expert opinion
	• Over-triage (total and all subgroups, with the exception of two-steps red sub-group, and one- and two-step black sub-groups)	• START and CTAS (two-step triage)	
	• Under-triage (total and all subgroups, with the exception of two-steps red sub-group, and one- and two-step black sub-groups)		
	• Under-triage (red classified as black)		
	• Under-triage (red classified as yellow)		
Lima et al. (45)	• Accuracy (total)	• START	Not reported
Loth et al. (36)	• Accuracy (total)	• START with training in triage before training	Not reported
		• START with training in triage after training	
		• START with training in transportation before training	
		• START with training in transportation after training	
McCoy et al. (7)	• Accuracy (total)	• START use by educator/technician/other	Not reported
		• START use by EMT/paramedics	
		• START use by nurses	
		• START use by pharmacists	
		• START use by physicians	
McElroy et al. (49)	• Accuracy (total)	• START	Not reported
	• Over-triage (total)		
	• Under-triage (total)		
Mills et al. (50)	• Accuracy (total)	• START using virtual reality	Not reported
		• START using live simulation	
Navin et al. (38)	• Accuracy (total)	• START	Not reported
	• Over-triage (total)	• Sacco Triage Method	
	• Under-triage (total)		
Risavi et al. (8)	• Accuracy (sub-groups green, yellow, and red)	• START with written triage first	Not reported
	• Accuracy for moulage (mean number of patients triaged correctly) at 6 months (total)	• START with moulage triage first	
	• Accuracy for written scenario (mean number of patients triaged correctly) at baseline (total)	• START with written triage second	
	• Accuracy for written scenario (mean number of patients triaged correctly) at 6 months (total)	• START with moulage triage second	
	• Accuracy for moulage (mean number of patients triaged correctly) at baseline (total)	• START with moulage at baseline	
	• Over-triage (sub-groups green, yellow, and red)	• START with moulage at 6 months	
	• Under-triage (sub-groups green, yellow, and red)	• START with written scenario at baseline	
		• START with written scenario at 6 months	
Riza'I et al. (41)	• Accuracy (total)	• START with lecture method	Not reported
	• Over-triage (total)	• START with simulation method	
	• Under-triage (total)		
Sapp et al. (32)	• Accuracy (total)	• START performed by students from year of 2008	Expert opinion
	• Over-triage (total)	• START performed by students from year of 2009	
	• Under-triage (total)	• START performed by students from year of 2008 and 2009	
Schenker et al. (44)	• Accuracy (total and all sub-groups, with exception of total black and first responding ambulance subgroup black)	• START performed on victims exiting triage area	Not reported
	• Over-triage (total and sub-groups)	• START performed by first responding ambulance	
	• Under-triage (total and sub-groups)	• Sum of START performed on victims exiting triage area and by first responding ambulance (?)	
Silvestri et al. (9)	• Over-triage (total)	• START	Expert opinion
	• Under-triage (total)	• SALT	
Simoes et al. (24)	• Accuracy (total)	• START	Not reported
	• Over-triage (total)		
	• Under-triage (total)		
Wu et al. (43)	• Accuracy (total)	• START performed by medical staff before training	Not reported
		• START performed by medical staff after training	
		• START performed by medical staff with no prior training before training	
		• START performed by medical staff with no prior training after training	
		• START performed by medical staff with prior training before training	
		• START performed by medical staff with prior training after training	
		• START performed by individuals with no prior training before training	
		• START performed by individuals with no prior training after training	
		• START performed by non-medical with no prior training before training	
		• START performed by non-medical with no prior training after training	
		• START performed by non-medical with prior training before training	
		• START performed by non-medical with prior training after training	
		• START performed by participants with prior training before training	
		• START performed by participants with prior training after training	

* *Wireless Internet Information System for Medical Response in Disasters*.

** *Personal digital assistant*.

*** *Electronic triage tag*.

**** *Traditional paper technology*.

##### Diagnostic Properties

A summary of the various diagnostic outcomes assessed across the studies are provided in Table 4. As per the inclusion criteria, all of the studies reported at least one outcome related to the classification accuracy of START. All but two studies (34, 46) assessed the accuracy of START by comparing participants' performance (correctly matching of triage levels to a reference standard).

With the exception of two studies (9, 48), all studies measuring classification accuracy of participants performance reported the overall accuracy for all victims. In addition, some studies also reported the accuracy of participants' performance based on the triage subgroups of START (i.e., black, red, yellow, and green) (8, 25, 26, 30, 31, 35, 42, 44, 47, 48). Still within accuracy of participants performance, some studies teased out the proportion of patients over and under-triaged within the START triage subgroups (8, 9, 23–27, 29, 30, 32, 35, 37, 38, 41, 42, 44, 46, 47, 49). Only two studies reported on outcomes related to START diagnostic properties, such as specificity, sensitivity, positive and negative predictive values, or likelihood ratios (34, 46).

Lastly, the vast majority of included studies (*n* = 22) did not specify which prerequisite they used to measure classification accuracy (i.e., a reference standard). When specified, the reference standard was most commonly described as expert opinions (9, 22, 23, 30–33, 37, 42) followed by the Baxt and Upeniek criticality (34), and the modified Baxt criteria (46). From the nine studies using experts' opinions as the reference standard, five studies did not specify the background of the experts or how this consensus was determined (22, 23, 30, 31, 33).

## Discussion

Given the widespread use of START for the triage of victims in real-world MCI's, training simulations, as well as assessing educational interventions, this scoping review aimed at exploring and summarizing the existing literature related to the current state of knowledge regarding studies assessing the classification accuracy of START. Gaining a better understanding of the literature helped us to identify gaps in reporting that may hold implications for future studies. Through an extensive and systematic search of the literature, 32 studies assessing the classification accuracy of START were identified. These studies were conducted around the world, with the majority of the studies published in the last 10 years, indicating that knowledge about simulation strategies using START for triage is a global concern and growing field of research.

Over the years, the methods used for simulations has changed as technological advancements occurred. For example, computer simulations replaced the early text-based paper exercises, and live simulations with actors have more recently been replaced by virtual reality technology. Studies included in our review employed different types of simulation technologies and, despite technological advancements, some of the most recently published studies employed technologies ranging from basic text-based exercises to the more advanced ones. This may be attributable to the high cost of using more advanced technologies during simulations, and the paucity of funding opportunities for disaster research within the research ecosystem. Although simulation can be effective at preparing individuals and systems to effectively deal with MCIs, it comes at a price. Different types of simulation technologies have different costs aggregated to them including training, equipment and systems, technicians, laboratory setup, maintenance and so on. In fact, the elevated costs of many simulation technologies has been a key criticism of medical training using simulation (51, 52). Therefore, it is reasonable that researchers developing MCI studies using simulation consider their population needs, available resources and return on investment to determine which type of technology they will study and adopt.

Other common themes arose when reviewing the articles, one of which was the reporting and implementation of the simulation. For the most part, studies provided satisfactory details regarding how the simulation exercises were conducted; however, the establishment of more systematic reporting is warranted. As discussed below, many studies lacked information that should be included in articles involving MCI simulation for them to be transparent, reproducible, and usable (53–55).

This review found that some important details regarding the methodologies of the studies and classification accuracy assessment were inconsistently reported across the literature. Approximately a third of the studies assessing the classification accuracy of START failed to report the type of MCI from which the victims were being triaged. Almost half of the studies did not specify the source of disaster scenarios—whether or not the MCI was based on a real event or created by the research staff, healthcare professionals, or disaster medicine experts. In many studies using live simulation, it was unclear if the mock victims had previous training on how to simulate clinical conditions or how these mock victims were prepared (e.g., use of make-up). At this time, it unclear whether the complexity of the disaster or MCI affects the classification accuracy of disaster triage, but this might be worth exploring in future studies.

Another common theme explored in this study was the reporting regarding the assessors of START and their experiences. It was not surprising that the majority of studies assessed the classification accuracy of paramedic/EMS providers to apply START; however, it was perhaps a little surprising that students (including paramedical, nursing, and medical) were the second most common assessors of START across the literature. It is not clear why this is the case. It could be that studies assessing novel technologies for simulations or triage methods may see students as a population of participants more available, willing and able to embrace novel technologies. In addition, students are more likely to lack any prior experience in disaster triage or START, allowing researchers to assess the impact of training or educational interventions on START classification accuracy.

A fundamental methodological bias associated with this literature is a lack of transparency which impacts the trustworthiness of the science. More than a third of the studies did not state if there was any potential conflict of interest. Over two-thirds did not state if there was any funding source. In addition, several studies did not acknowledge any limitations to the study, and the ones acknowledging them overlooked or reduced to simplistic and minimally relevant themes (e.g., single institution study or small sample size) (56). With respect to the assessment of the classification accuracy of START, while the majority of the studies reported overall accuracy, a third of them did not report under- and over- triage. It is vital for studies assessing triage accuracy to provide a full assessment of the classification accuracy of START. Beneficial triage decisions direct victims to the most appropriate hospitals, resulting in lower mortality and better resource allocation (57).

Yet, one of the most concerning issues we found in this review exploring the current state of knowledge of studies assessing the classification accuracy of the START system was that two-thirds of the studies completely lacked details regarding the reference standard to which START was being compared. When a reference standard was reported, the most common was expert opinion, although details regarding the credentials of the experts were not provided. The traditional classification accuracy paradigm is based on studies that compare the results of the system under evaluation (index system) with the results of a reference standard, and it is regarded as the soundest method to determine the classification accuracy of the system or measure participants' performance. To appraise the classification accuracy of the index test, its results are compared with the results of the reference standard; subsequently indicators of accuracy can be determined. The reference standard is therefore an important determinant of the classification accuracy. From a theoretical perspective the use of an appropriate reference standard is critical and the lack of information regarding it impacts the confidence readers have in research findings.

### Strengths and Limitations

We aimed at using precise and transparent review methods when conducting (16, 17) and reporting this scoping review (18). A comprehensive approach using several appropriate databases without language restrictions improved the rigor of the review. Consistent with the purpose of a scoping review, we expanded the literature search from January 1983 until March 2020, so that more literature sources could be identified, and findings could truly reflect the state of knowledge. The search words were selected by the researchers and refined by an expert health librarian. In addition, the reference lists of the included articles were forward searched. To reduce the risk of selection bias, this review utilized two independent reviewers to assess and identify potential eligible studies. Lastly, the use of Refworks and Covidence software supported meticulous documentation of screening decisions.

There were, however, some limitations of this scoping review. First, since this review did not pursue quality appraisal, we were not able to speak of the quality of the studies in the field assessing the classification accuracy of START, which could have resulted in inclusion of studies with comprised research quality and incomplete synthesis. Therefore, it is recommended that the findings should be used with caution and applied in research and practice after careful scrutiny. Second, 87.5% (*n* = 28) of the reviewed studies originated from developed countries which limits the extrapolation of findings to low- and middle-income countries. Third, the results of this scoping review may have been impacted by selective reporting within the included studies. While contacting the study authors could have helped clarify aspects of the simulation, triage assessment, or accuracy outcomes that were unclear or not reported, the objective of this review was to provide an assessment of studies assessing START accuracy based on what is reported in the available literature. Lastly, as with any review, there is a risk of publication bias, particularly among studies assessing the impact of novel interventions on triage classification accuracy.

## Conclusion

Studies included in this scoping review provided satisfactory details on how their simulations were conducted. However, we found there is room for improvement in view of insufficient information regarding location where simulation exercises were performed, the type of disaster they were simulating, the source of the MCI event, the characteristics of the victims, whether or not participants had any prior experience with MCI triage, and potential source of bias. To further improve simulation-based assessment of triage systems, it is important that stakeholders are mindful of the complexity of subsystem interactions. It is recommended that if simulations are used for assessment purposes, they should be based in a systematic appreciation of the whole system. Future research could be more explicit about the knowledge upon which simulation training is based to allow for description of core theoretical and operational definitions, identification of the function of each component, promotion of similar construct measurement, reporting of findings in a common language, as well as replication and comparison of findings across studies. We recommend the use of reporting guidelines such as the “reporting guidelines for health care simulation research: extensions to the CONSORT and STROBE statements” (11). In particular, incomplete reporting of the reference standards and accuracy needs to be addressed and reported in future studies.

We recommend the development of a systematic review with meta-synthesis to assess overall accuracy, rate of under-triage, and rate of over-triage using the START method, as well as to obtain specific rates of accuracy for each of the four START categories: red, yellow, green, and black. A systematic review with meta-synthesis will allow the combination of results ensuring reliability across a number of studies, while assessing and minimizing bias. As a result, reliable and scientifically derived findings can be obtained for research and clinical practice.

## Data Availability Statement

The original contributions presented in the study are included in the article/Supplementary Material, further inquiries can be directed to the corresponding author.

## Author Contributions

UDW: research conceptualization, design of the research methodology, data curation, evidence screening, data extraction, data analysis, project administration, writing and editing the research protocol, and writing and editing the final manuscript. SWK: research conceptualization, design of the research methodology, data curation, evidence screening, data extraction, project administration, writing and editing the research protocol, and writing and editing the final manuscript. BHR: research conceptualization, design of the research methodology, funding acquisition, research supervision, writing and editing the research protocol, and writing and editing the final manuscript. SC: design of the research methodology and writing and editing the final manuscript. JMF: research conceptualization, design of the research methodology, funding acquisition, data analysis, writing and editing the research protocol, and writing and editing the final manuscript. All authors contributed to the article and approved the submitted version.

## Funding

Scoping and Systematic Review Grant from the Emergency Strategic Clinical Network (ESCN) at Alberta Health Services and the Emergency Medicine Research Group (EMeRG) in the Department of Emergency Medicine at the University of Alberta. BHR's research is supported by a Scientific Director's Grant (SOP 168483) from the Canadian Institutes of Health Research (CIHR; Ottawa, ON). The funders had no role in the design, implementation, analysis, and write-up of the study.

## Author Disclaimer

The content hereof is the sole responsibility of the authors and does not necessarily represent the official views of the funding agencies.

## Conflict of Interest

JMF is CEO and Founder of Stat59. The remaining authors declare that the research was conducted in the absence of any commercial or financial relationships that could be construed as a potential conflict of interest.

## Publisher's Note

All claims expressed in this article are solely those of the authors and do not necessarily represent those of their affiliated organizations, or those of the publisher, the editors and the reviewers. Any product that may be evaluated in this article, or claim that may be made by its manufacturer, is not guaranteed or endorsed by the publisher.

## References

[1] Tonkin L. Triage: multiple casualty incidents. Aust J Emerg Care. (1997) 4:18–21.

[2] Douglas N Leverett J Paul J Gibson M Pritchard J Brouwer K . Performance of first aid trained staff using a modified START triage tool at achieving appropriate triage compared to a physiology-based triage strategy at Australian mass gatherings. Prehospital Disaster Med. (2020) 35:184–8. 10.1017/S1049023X20000102 31983350

[3] Arnold T Cleary V Groth S Hook R Jones D Super G. START. Newport Beach, CA: Newport Beach Fire and Marine Department (1994).

[4] Garner A Harrison K Lee A Schultz CH. Comparative analysis of multiple-casualty incident triage algorithms. Ann Emerg Med. (2001) 38:541–8. 10.1067/mem.2001.119053 11679866

[5] Wallis L. START is not the best triage stategy. Br J Sports Med. (2002) 36:473. 10.1136/bjsm.36.6.473 12453848 PMC1724560

[6] Bazyar J Farrokhi M Khankeh H. Triage systems in mass casualty incidents and disasters: a review study with a worldwide approach. Open Access Maced J Med Sci. (2019) 7:482–94. 10.3889/oamjms.2019.119 30834023 PMC6390156

[7] McCoy CE Alrabah R Weichmann W Langdorf MI Ricks C Chakravarthy B . Feasibility of telesimulation and Google Glass for mass casualty triage education and training. West J Emerg Med. (2019) 20:512–9. 10.5811/westjem.2019.3.40805 31123554 PMC6526878

[8] Risavi BL Lee W Terrell MA Holsten DL. Prehospital mass-casualty triage training-written versus moulage scenarios: how much do EMS providers retain? Prehospital Disaster Med. (2013) 28:251–6. 10.1017/S1049023X13000241 23507083

[9] Silvestri S Field A Mangalat N Weatherford T Hunter C McGowan Z . Comparison of START and SALT triage methodologies to reference standard definitions and to a field mass casualty simulation. Am J Disaster Med. (2017) 12:27–33. 10.5055/ajdm.2017.0255 28822212

[10] Franc JM Kirkland SW Wisnesky UD Campbell S Rowe BH. METASTART: a systematic review and meta-analysis of the diagnostic accuracy of the Simple Triage and Rapid Treatment (START) algorithm for disaster triage. Prehospital Disaster Med. (2021) 2021:1–11. 10.1017/S1049023X2100131X 34915954

[11] Cheng A Kessler D Mackinnon R Chang TP Nadkarni VM Hunt EA . Reporting guidelines for health care simulation research: extensions to the CONSORT and STROBE statements. Adv Simul. (2016) 1:25. 10.1186/s41077-016-0025-y 29449994 PMC5806464

[12] Germini F Marcucci M Heath T Mbuagbaw L Thabane L Worster A . Quality of reporting in abstracts of RCTs published in emergency medicine journals: a systematic survey of the literature suggests we can do better. Emerg Med J. (2019). 10.1136/emermed-2019-208629 31694858

[13] Monks T Currie CSM Onggo BS Robinson S Kunc M Taylor SJE. Strengthening the reporting of empirical simulation studies: introducing the STRESS guidelines. J Simul. (2019) 13:55–67. 10.1080/17477778.2018.1442155

[14] Nawijn F Ham WHW Houwert RM Groenwold RHH Hietbrink F Smeeing DPJ. Quality of reporting of systematic reviews and meta-analyses in emergency medicine based on the PRISMA statement. BMC Emerg Med. (2019) 19:1–18. 10.1186/s12873-019-0233-6 30744570 PMC6371507

[15] Zhang X Lhachimi SK Rogowski WH. Reporting quality of discrete event simulations in healthcare—results from a generic reporting checklist. Value Health. (2020) 23:506–14. 10.1016/j.jval.2020.01.005 32327168

[16] Arksey H O'Malley L. Scoping studies: towards a methodological framework. Int J Soc Res Methodol. (2005) 8:19–32. 10.1080/1364557032000119616

[17] Levac D Colquhoun H O'Brien KK. Scoping studies: advancing the methodology. Implement Sci. (2010) 5:69–77. 10.1186/1748-5908-5-69 20854677 PMC2954944

[18] Tricco AC Lillie E Zarin W O'Brien KK Colquhoun H Levac D . PRISMA extension for scoping reviews (PRISMA-ScR): checklist and explanation. Ann Intern Med. (2018) 169:467–73. 10.7326/M18-0850 30178033

[19] Moher D Liberati A Tetzlaff J Altman DG. Preferred reporting items for systematic reviews and meta-analyses: the PRISMA statement. BMJ. (2009) 339:b2535. 10.1136/bmj.b2535 19622551 PMC2714657

[20] Shaluf IM. Disaster types. Disaster Prev Manag. (2007) 16:704–17. 10.1108/09653560710837019

[21] Popay J Roberts H Snowden A Petticrew M Arai L Britten N . Guidance on the Conduct of Narrative Synthesis in Systematic Reviews: Final Report. Swindon: ESRC Research Methods Programme (2006).

[22] Buono CJ Lyon J Huang R Liu F Brown S Killeen JP . Comparison of mass casualty incident triage acuity status accuracy by traditional paper method, electronic tag, and provider PDA algorithm. Ann Emerg Med. (2007) 50:S12–S3. 10.1016/j.annemergmed.2007.06.068

[23] Crews CM. Disaster Response: Efficacy of Simple Triage and Rapid Treatment in Mass Casualty Incidents. Master's thesis. Long Beach, CA: California State University (2008).

[24] Simões RL Duarte Neto C Maciel GSB Furtado TP Paulo DNS. Atendimento pré-hospitalar à múltiplas vítimas com trauma simulado [Prehospital care to trauma victmis with multiple simulated]. Rev Col Bras Cir. (2012) 39:230–7. 10.1590/S0100-69912012000300013 22836574

[25] Ellebrecht N Latasch L. Vorsichtung durch Rettungsassistenten auf der Großübung SOGRO MANV 500: Eine vergleichende Analyse der Fehleinstufungen. Paramedic triage during a mass casualty incident exercise: a comparative analysis of inappropriate triage level assignments. Notfall Rettungsmed. (2012) 15:58. 10.1007/s10049-011-1477-1

[26] Badiali S Giugni A Marcis L. Testing the START triage protocol: can it improve the ability of nonmedical personnel to better triage patients during disasters and mass casualties incidents? Disaster Med Public Health Prep. (2017) 11:305–9. 10.1017/dmp.2016.151 28065200

[27] Ingrassia PL Ragazzoni L Carenzo L Colombo D Gallardo AR Corte FD. Virtual reality and live simulation: a comparison between two simulation tools for assessing mass casualty triage skills. Eur J Emerg Med. (2015) 22:121–7. 10.1097/MEJ?0000000000000132 24841770

[28] Jain T Sibley A Stryhn H Hubloue I. Comparison of unmanned aerial vehicle technology-assisted triage versus standard practice in triaging casualties by paramedic students in a mass-casualty incident scenario. Prehospital Disaster Med. (2018) 33:375–80. 10.1017/S1049023X18000559 30001765

[29] Khan K. Tabletop exercise on mass casualty incident triage, does it work? Health Sci J. (2018) 12:1–6. 10.21767/1791-809X.100056620066638

[30] Lee JS Franc JM. Impact of a two-step emergency department triage model with START, then CTAS, on patient flow during a simulated mass-casualty incident. Prehospital Disaster Med. (2015) 30:390–6. 10.1017/S1049023X15004835 26105567

[31] Bolduc C Maghraby N Fok P Luong TM Homier V. Comparison of electronic versus manual mass-casualty incident triage. Prehospital Disaster Med. (2018) 33:273–8. 10.1017/S1049023X1800033X 29661267

[32] Sapp RF Brice JH Myers JB Hinchey P. Triage performance of first-year medical students using a multiple-casualty scenario, paper exercise. Prehospital Disaster Med. (2010) 25:239–45. 10.1017/S1049023X00008104 20586018

[33] Ferrandini-Price M Escribano Tortosa D Nieto Fernandez-Pacheco A Perez Alonso N Cerón Madrigal JJ Melendreras-Ruiz R . Comparative study of a simulated incident with multiple victims and immersive virtual reality. Nurse Educ Today. (2018) 71:48–53. 10.1016/j.nedt.2018.09.006 30241022

[34] Challen K Walter D. Major incident triage: comparative validation using data from 7th July bombings. Injury. (2013) 44:629–33. 10.1016/j.injury.2012.06.026 22877789

[35] Arshad FH Williams A Asaeda G Isaacs D Kaufman B Ben-Eli D . A modified simple triage and rapid treatment algorithm from the New York City (USA) fire department. Prehospital Disaster Med. (2015) 30:199–204. 10.1017/S1049023X14001447 25687598

[36] Loth S Cote AC Shaafi Kabiri N Bhangu JS Zumwalt A Moss M . Improving triage accuracy in first responders: measurement of short structured protocols to improve identification of salient triage features. World Med Health Policy. (2019) 11:163–76. 10.1002/wmh3.306

[37] Curran-Sills G Franc JM. A pilot study examining the speed and accuracy of triage for simulated disaster patients in an emergency department setting: comparison of a computerized version of Canadian Triage Acuity Scale (CTAS) and Simple Triage and Rapid Treatment (START) methods. CJEM. (2017) 19:364–71. 10.1017/cem.2016.386 27788698

[38] Navin DM Sacco WJ Waddell R. Operational comparison of the simple triage and rapid treatment method and the sacco triage method in mass casualty exercises. J Trauma Nurs. (2010) 69:215–25. 10.1097/TA.0b013e3181d74ea4

[39] Izumida K Kato R Shigeno H. A triage training system considering cooperation and proficiency of multiple trainees. In: Yoshino T, Yuizono T, Zurita G, Vassileva J, editors. Lecture Notes in Computer Science. Cham: Springer (2017). p. 68–83.

[40] Ingrassia PL Ragazzoni L Tengattini M Carenzo L Corte FD. Nationwide program of education for undergraduates in the field of disaster medicine: development of a core curriculum centered on blended learning and simulation tools. Prehospital Disaster Med. (2014) 29:508–15. 10.1017/S1049023X14000831 25155942

[41] Riza'i A Ade WRA Albar I Sulitio S Muharris R. Teaching start triage: a comparison of lecture and simulation methods. Adv Sci Lett. (2018) 24:6890–2. 10.1166/asl.2018.1287424841770

[42] Ingrassia PL Colombo D Barra FL Carenzo L Della Corte F Franc J. Impact of training in medical disaster management: a pilot study using a new tool for live simulation. Emergencias. (2013) 25:459–66.

[43] Wu Y-L Shu C-C Chung C-C. A simple method for pre-hospital dispatcher-aided consciousness assessment in trauma patients. J Emerg Med Taiwan. (2005) 7:69–77. 10.30018/JECCM.199906.0002

[44] Schenker JD Goldstein S Braun J Werner A Buccellato F Asaeda G . Triage accuracy at a multiple casualty incident disaster drill: the Emergency Medical Service, Fire Department of New York City experience. J Burn Care Res. (2006) 27:570–5. 10.1097/01.BCR.0000235450.12988.27 16998387

[45] Lima DS De-Vasc Oncelos IF Queiroz EF Cunha TA Dos-Santos VS Freitas JG . Multiple victims incident simulation: training professionals and university teaching. Rev Col Bras Cir. (2019) 46:e20192163. 10.1590/0100-6991e-20192163 31389523

[46] Kahn CA Schultz CH Miller KT Anderson CL. Does START triage work? An outcomes assessment after a disaster. Ann Emerg Med. (2009) 54:424–30.e1. 10.1016/j.annemergmed.2008.12.035 19195739

[47] Ersoy N Akpinar A. Triage decisions of emergency physicians in Kocaeli and the principle of justice. Ulusal Travma ve Acil Cerrahi Dergisi. (2010) 16:203–9. 20517743

[48] Djalali A Carenzo L Ragazzoni L Corte FD Ingrassia PL Azzaretto M . Does hospital disaster preparedness predict response performance during a full-scale exercise? A pilot study. Prehospital Disaster Med. (2014) 29:441–7. 10.1017/S1049023X1400082X 25093456

[49] McElroy JA Steinberg S Keller J Falcone RE. Operation continued care: a large mass-casualty, full-scale exercise as a test of regional preparedness. Surgery. (2019) 166:587–92. 10.1016/j.surg.2019.05.045 31447104

[50] Mills B Dykstra P Hansen S Miles A Rankin T Hopper L . Virtual reality triage training can provide comparable simulation efficacy for paramedicine students compared to live simulation-based scenarios. Prehosp Emerg Care. (2019) 24:525–36. 10.1080/10903127.2019.1676345 31580178

[51] Hippe DS Umoren RA McGee A Bucher SL Bresnahan BW. A targeted systematic review of cost analyses for implementation of simulation-based education in healthcare. SAGE Open Med. (2020) 8:2050312120913451. 10.1177/2050312120913451 32231781 PMC7082864

[52] Mücke U Grigull L Sänger B Berndt LP Wittenbecher H Schwarzbard C . Introducing low-cost simulation equipment for simulation-based team training. Clin Simul Nurs. (2020) 38:18–22. 10.1016/j.ecns.2019.09.001

[53] Munafò MR Nosek BA Bishop DVM Button KS Chambers CD Percie du Sert N . A manifesto for reproducible science. Nat Hum Behav. (2017) 1:0021. 10.1038/s41562-016-0021 33954258 PMC7610724

[54] Moher D. Reporting guidelines: doing better for readers. BMC Med. (2018) 16:233. 10.1186/s12916-018-1226-0 30545364 PMC6293542

[55] Hoffmann TC Oxman AD Ioannidis JP Moher D Lasserson TJ Tovey DI . Enhancing the usability of systematic reviews by improving the consideration and description of interventions. BMJ. (2017) 358:j2998. 10.1136/bmj.j2998 28729459

[56] Ross PT Bibler Zaidi NL. Limited by our limitations. Perspect Med Educ. (2019) 8:261–4. 10.1007/s40037-019-00530-x 31347033 PMC6684501

[57] Najafi Z Abbaszadeh A Zakeri H Mirhaghi A. Determination of mis-triage in trauma patients: a systematic review. Eur J Trauma Emerg Surg. (2019) 45:821–39. 10.1007/s00068-019-01097-2 30798344

