# Supplementary material for: A Qualitative Assessment of Studies Evaluating the Classification Accuracy of Personnel Using START in Disaster Triage: A Scoping Review
Source: Front Public Health. 2022 Feb 24;10:676704. doi: 10.3389/fpubh.2022.676704 (PMC8907512; doi:10.3389/fpubh.2022.676704)
Supplement: Supplementary file 2 [file Table_2.DOCX]

**Supplementary file 2. SEARCH STRATEGIES**

**Ovid MEDLINE(R) ALL <1946 to March 20, 2020>**

| **#** | **Search Statement** | **Results** |
| --- | --- | --- |
| 1 | Start triage.mp. | 28 |
| 2 | (Simple Triage and Rapid).mp. | 87 |
| 3 | (start and triage*).ti. ["jumpstart" not searched because it is the pediatric version of START] | 31 |
| 4 | 1 or 2 or 3 | 108 |
| 5 | ("mass casualt*" or (mass adj3 incident*) or disaster*).ti,ab,kw. or exp *Mass Casualty Incidents/ or exp *Disasters/ | 59960 |
| 6 | triag*.ti. | 5328 |
| 7 | 5 and 6 | 490 |
| 8 | (MSK or (STOPP adj2 START) or "back tool").mp. or (sydney or "back screen*").ti,ab. | 11745 |
| 9 | 4 or 7 | 537 |
| 10 | 9 not 8 | 532 |
| 11 | limit 10 to yr="1983 -Current" | 512 |

**Embase <1974 to 2020 March 20>**

| **#** | **Search Statement** | **Results** |
| --- | --- | --- |
| 1 | mSTART.mp. ["modified START" not included because all relevant records in Medline also included in retrieval for lines 2 and 3] | 15 |
| 2 | "Simple Triage and Rapid".mp. | 89 |
| 3 | ((START adj5 triage*) or (START adj2 tool)).mp. ["JUMPSTART" not searched because it is the pediatric version of START] | 368 |
| 4 | 1 or 2 or 3 | 394 |
| 5 | ("mass casualt*" or (mass adj3 incident*) or disaster*).ti,ab,kw. or *disaster/ or *mass disaster/ or *natural disaster/ or *rescue work/ or *disaster medicine/ | 38428 |
| 6 | triag*.ti. | 7187 |
| 7 | 5 and 6 | 489 |
| 8 | 4 or 7 | 799 |
| 9 | sydney.ti,ab,kw. or ("back screen*" or "back tool*" or MSK or (START adj1 STOPP)).mp. | 18107 |
| 10 | 8 not 9 | 641 |
| 11 | limit 10 to yr="1983 -Current" [date START was introduced. Limit used to prevent retrieval pre-1983 articles relating to mass casualty and triage, which might also contain the word "start"] | 624 |

**Global Health <1973 to 2020 Week 11>**

| # | Search Statement | Results |
| --- | --- | --- |
| 1 | mSTART.mp. ["modified START" not included because all relevant records in Medline also included in retrieval for lines 2 and 3] | 1 |
| 2 | "Simple Triage and Rapid".mp. | 7 |
| 3 | ((START adj5 triage*) or (START adj2 tool)).mp. ["JUMPSTART" not searched because it is the pediatric version of START] | 15 |
| 4 | 1 or 2 or 3 | 18 |
| 5 | ("mass casualt*" or (mass adj3 incident*) or disaster*).ti,ab. | 6512 |
| 6 | triag*.ti. | 488 |
| 7 | 5 and 6 | 46 |
| 8 | 4 or 7 | 59 |
| 9 | sydney.ti,ab. or ("back screen*" or "back tool*" or MSK or (START adj1 STOPP)).mp. | 3152 |
| 10 | 8 not 9 | 55 |
| 11 | limit 10 to yr="1983 -Current" [date START was introduced. Limit used to prevent retrieval pre-1983 articles relating to mass casualty and triage, which might also contain the word "start"] | 55 |

**Compendex for 1884-2020 Searched March 24, 2020 Results =57**

((((((start and triage)) WN ALL) OR ((triag*) WN TI)) AND (((disaster* or "mass casualt*" or "mass injur*" or "mass incident*" or "building collapse" or hurricane* or storm surge* or tsunami* or earthquake* or "earth quake*" or "mass evacuat*" or tornado* or "wildfire*" or MCI "mud slide*")) WN ALL)) NOT (("back screen*" or "back pain" or "back tool*" or msk or sydney or "stopp start" or "start stopp") WN ALL))**CINAHL with full-text Searched March 23, 2020**

| \| # \| Query \| Limiters/Expanders \| Results \| \| --- \| --- \| --- \| --- \| \| S1 \| ( mstart or "Simple Triage and Rapid" ) OR ( START N5 triage* or START N2 tool ) \| Expanders - Apply equivalent subjects  Search modes - Find all my search terms \| 258 \| \| S2 \| AB ("mass casualt*" or (mass N3 incident*) or disaster* \| Expanders - Apply equivalent subjects  Search modes - Find all my search terms \| 9,223 \| \| S3 \| TI ("mass casualt*" or (mass N3 incident*) or disaster* \| Expanders - Apply equivalent subjects  Search modes - Find all my search terms \| 9,264 \| \| S4 \| (MM "Disasters+") OR (MM "Fires+") OR (MM "Mass Casualty Incidents") OR (MM "Natural Disasters") OR (MM "Emergency Evacuation") \| Expanders - Apply equivalent subjects  Search modes - Find all my search terms \| 26,230 \| \| S5 \| TI triag* \| Expanders - Apply equivalent subjects  Search modes - Find all my search terms \| 4,710 \| \| S6 \| S2 OR S3 OR S4 \| Expanders - Apply equivalent subjects  Search modes - Find all my search terms \| 32,104 \| \| S7 \| S5 AND S6 \| Expanders - Apply equivalent subjects  Search modes - Find all my search terms \| 318 \| \| S8 \| ( sydney or "back N3 screen*" or "back pain" or "start back" or "back tool" or msk ) OR ( "START STOPP" or "STOPP START" ) \| Expanders - Apply equivalent subjects  Search modes - Find all my search terms \| 45,589 \| \| S9 \| S1 OR S7 \| Expanders - Apply equivalent subjects  Search modes - Find all my search terms \| 540 \| \| S10 \| s9 NOT s8 \| Expanders - Apply equivalent subjects  Search modes - Find all my search terms \| 403 \| \| S11 \| s9 NOT s8 \| Limiters - Published Date: 19830101-20201231  Expanders - Apply equivalent subjects  Search modes - Find all my search terms \| 400 \| |
| --- | --- | --- | --- | --- | --- | --- | --- | --- | --- | --- | --- | --- | --- | --- | --- | --- | --- | --- | --- | --- | --- | --- | --- | --- | --- | --- | --- | --- | --- | --- | --- | --- | --- | --- | --- | --- | --- | --- | --- | --- | --- | --- | --- | --- | --- | --- | --- | --- |

**PROSPERO Searched March 24, 2020**

LineSearch forHits

#1mstart or (start and triag*) or "simple triage and rapid" 17

#2(triag*):TI 45

#3mass or disaster* or tsunami* or "tidal wave*" or tornado* or hurricane* or earthquake* or "earth quake" or flood* or "mud slide*" or "building collaps*" 3872

#4#2 AND # 36

#5#1 OR #4 23

**Cochrane Library Searched March 23, 2020**

ID Search Hits

#1 (mstart or "simple triage and rapid"):ti,ab,kw OR ((start and triag*)):ti (Word variations have been searched) 14

#2 ("mass casualt*" or "mass incident*" or disaster*):ti,ab,kw AND (triag*):ti (Word variations have been searched) 25

#3 MeSH descriptor: [Disasters] explode all trees 1473

#4 MeSH descriptor: [Mass Casualty Incidents] this term only 22

#5 #3 or #4 1473

#6 (triag*):ti 478

#7 #5 and #6 26

#8 #1 or #2 or #7 40

**ProQuest Dissertations and Theses Global Searched March 24, 2020**

noft(mstart OR (start AND triag*) OR "simple triage and rapid") OR (ti(triag* ) AND noft(disaster* or "mass casualt*" or "mass injur*" or "mass incident*" or "building collapse" or hurricane* or storm surge* or tsunami* or earthquake* or "earth quake*" or "mass evacuat*" or tornado* or "wildfire*" or "mud slide*"))

**SCOPUS Searched March 24, 2020**

( TITLE-ABS-KEY ( mstart OR "simple triage and rapid" OR start W/5 triag* ) ) OR ( ( TITLE ( triag* ) ) AND ( TITLE-ABS-KEY ( ( disaster* OR "mass casualt*" OR "mass injur*" OR "mass incident*" OR "building collapse" OR hurricane* OR storm AND surge* OR tsunami* OR earthquake* OR "earth quake*" OR "mass evacuat*" OR tornado* OR "wildfire*" OR "mud slide*" ) ) ) ) AND NOT ( TITLE-ABS-KEY ( ( msk OR "back screen*" OR "back tool*" OR start W/2 stopp OR sydney ) ) ) AND ( LIMIT-TO ( PUBYEAR , 2020 ) OR LIMIT-TO ( PUBYEAR , 2019 ) OR LIMIT-TO ( PUBYEAR , 2018 ) OR LIMIT-TO ( PUBYEAR , 2017 ) OR LIMIT-TO ( PUBYEAR , 2016 ) OR LIMIT-TO ( PUBYEAR , 2015 ) OR LIMIT-TO ( PUBYEAR , 2014 ) OR LIMIT-TO ( PUBYEAR , 2013 ) OR LIMIT-TO ( PUBYEAR , 2012 ) OR LIMIT-TO ( PUBYEAR , 2011 ) OR LIMIT-TO ( PUBYEAR , 2010 ) OR LIMIT-TO ( PUBYEAR , 2009 ) OR LIMIT-TO ( PUBYEAR , 2008 ) OR LIMIT-TO ( PUBYEAR , 2007 ) OR LIMIT-TO ( PUBYEAR , 2006 ) OR LIMIT-TO ( PUBYEAR , 2005 ) OR LIMIT-TO ( PUBYEAR , 2004 ) OR LIMIT-TO ( PUBYEAR , 2003 ) OR LIMIT-TO ( PUBYEAR , 2002 ) OR LIMIT-TO ( PUBYEAR , 2001 ) OR LIMIT-TO ( PUBYEAR , 2000 ) OR LIMIT-TO ( PUBYEAR , 1999 ) OR LIMIT-TO ( PUBYEAR , 1998 ) OR LIMIT-TO ( PUBYEAR , 1996 ) OR LIMIT-TO ( PUBYEAR , 1994 ) OR LIMIT-TO ( PUBYEAR , 1991 ) OR LIMIT-TO ( PUBYEAR , 1990 ) OR LIMIT-TO ( PUBYEAR , 1986 ) OR LIMIT-TO ( PUBYEAR , 1980 ) )
